# Data from the analytical performance of the Abaxis Piccolo Xpress point of care analyzer in whole blood, serum, and plasma

**DOI:** 10.1016/j.dib.2017.11.006

**Published:** 2017-11-04

**Authors:** Kazunori Murata, Laurel Glaser, Mary Nardiello, Lakshmi V. Ramanathan, Dean C. Carlow

**Affiliations:** a Department of Laboratory Medicine, Memorial Sloan Kettering Cancer Center, New York, USA; b Department of Pathology and Laboratory Medicine, The Perelman School of Medicine at the University of Pennsylvania, Philadelphia, USA; c Department of Pathology and Laboratory Medicine, Children's Hospital of Philadelphia, Philadelphia, USA

**Keywords:** Clinical chemistry, Point-of care testing

## Abstract

The objective of this study was to examine the analytical performance of 14 comprehensive metabolic panel analytes on the Abaxis Piccolo Xpress® Point of Care analyzer in serum, plasma, and whole blood. A method comparison was performed on all three specimen types intended for use on the Piccolo Xpress®: serum, heparinized plasma, and whole blood. This data is also presented in Murata et al. (2015) [1]. This article includes the actual Bland-Altman bias plots of the difference in results obtained for analytes in the comprehensive metabolic panel from the Abaxis Piccolo Xpress and the comparison instrument, the Ortho Vitros.

**Specifications Table**

**Table:** 

Subject area	Laboratory medicine
More specific subject area	Point of Care
Type of data	Graph
How data was acquired	Photometric, enzymatic, ion selective electrodes,
Data format	Plotted raw data
Experimental factors	Specimens were collected into EDTA, plasma, or serum tubes. Plasma and serum tubes were centrifuged to separate out blood cells prior to chemical analysis
Experimental features	Serum, whole blood, and plasma were analyzed on both the Ortho Vitros and the Abaxis Piccolo for 14 analytes which comprise the comprehensive metabolic panel.
Data source location	Philadelphia, PA, and New York, NY
Data accessibility	Data with article
Related research article	Murata et al. [1].

**Value of the data**

● These are the raw data to support the summary published in Ref. [1].
● This data graphically show the biases between the Piccolo point of care device and the Ortho Vitros analyzer for each of the analytes in the comprehensive metabolic panel.
● This study is the first to examine the analytical performance of all three matrices approved by the FDA for analysis on the Abaxis Piccolo: whole blood, serum, and plasma.

## 1 Data

**Fig. 1:**
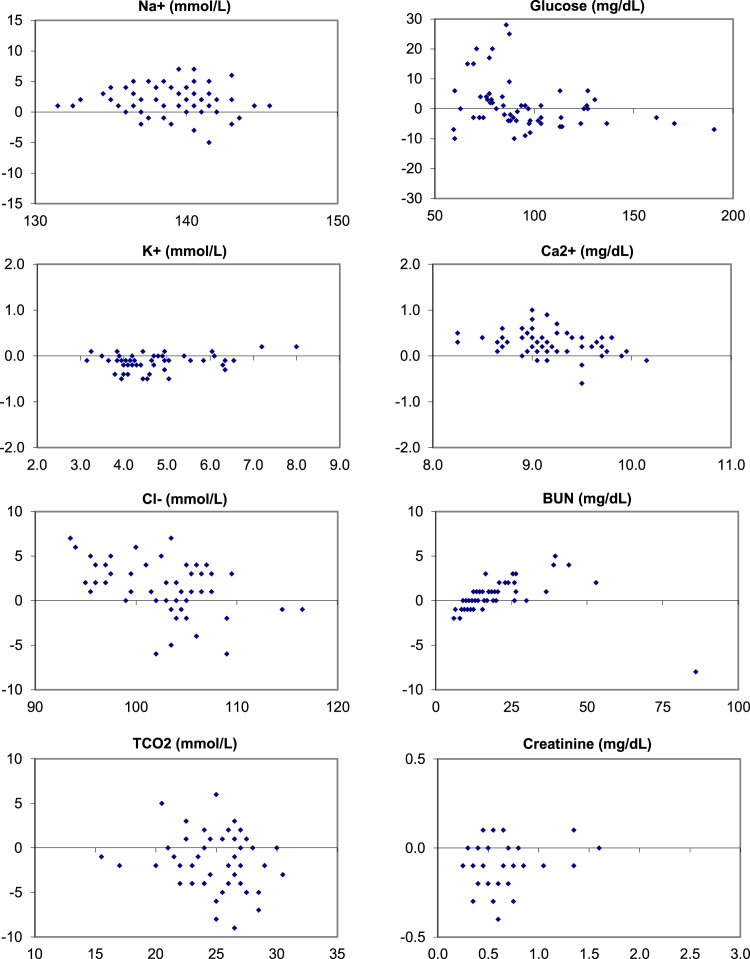

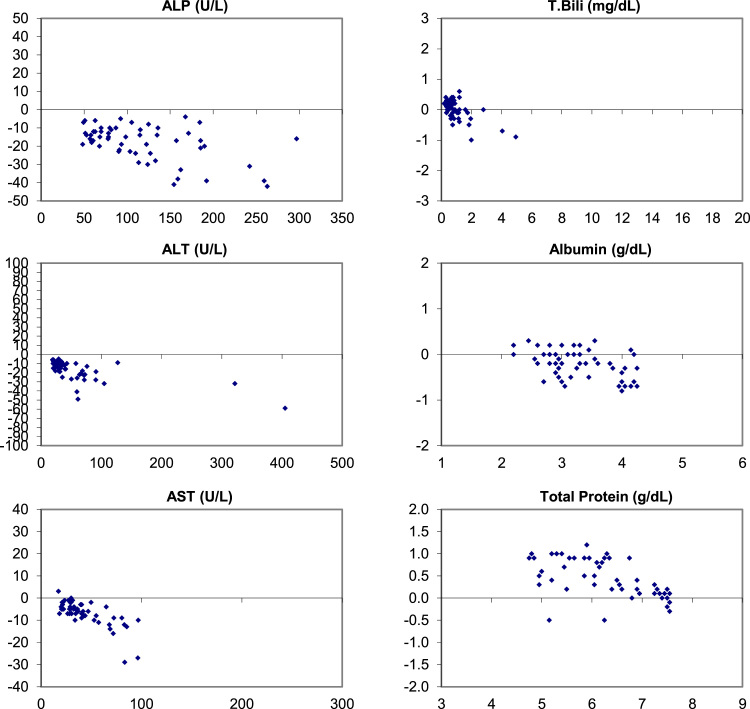
Bias (Bland-Altman) Plots of analytical bias between plasma specimens on the Ortho Vitros and whole blood specimens on the Abaxis Piccolo for all analytes in the comprehensive metabolic panel.

**Fig. 2:**
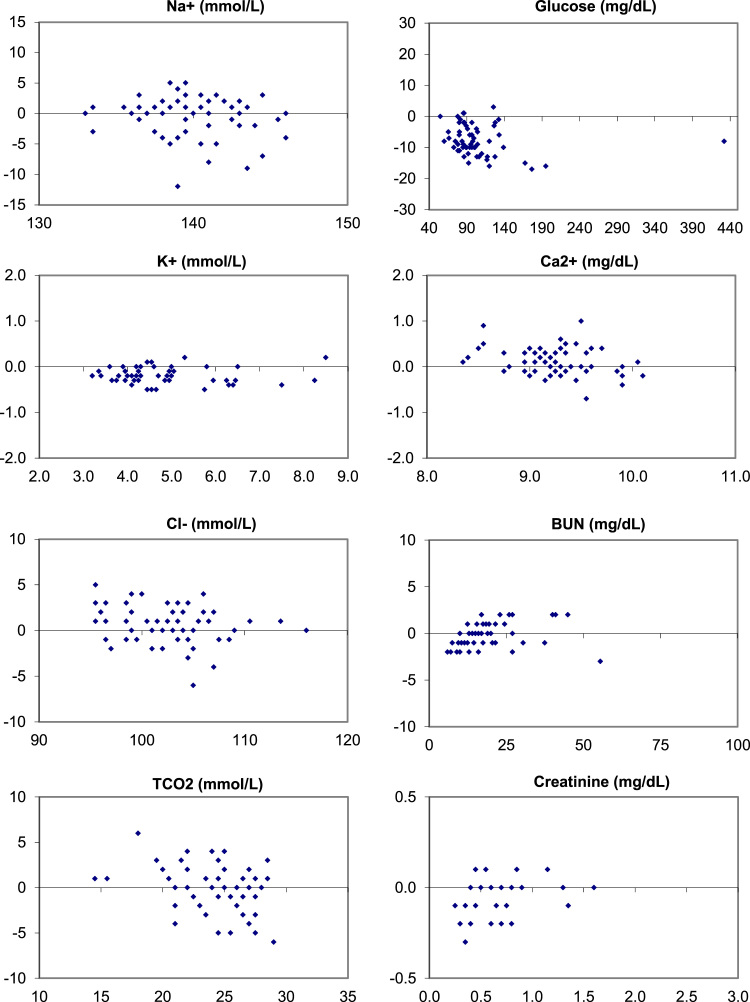

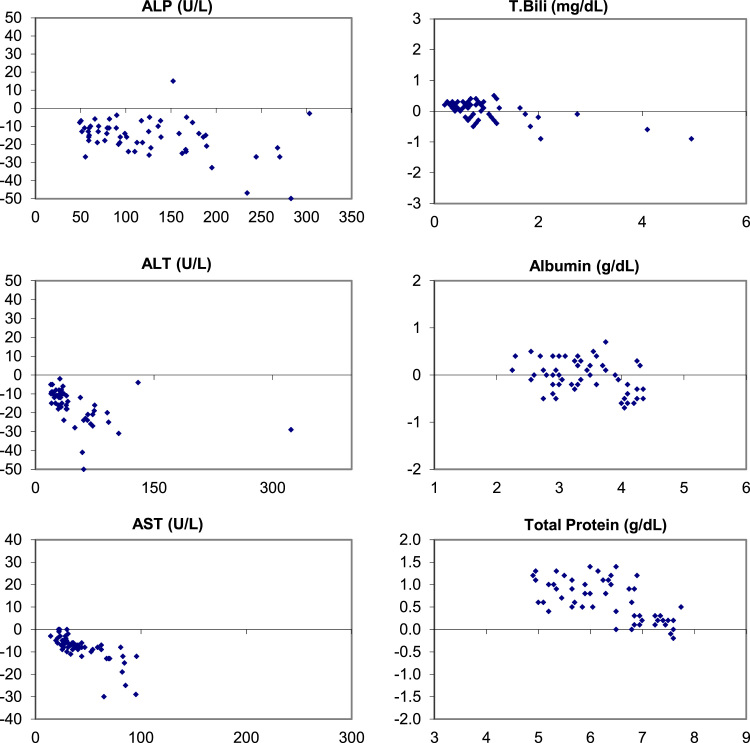
Bias (Bland-Altman) Plots of analytical bias between plasma specimens on the Ortho Vitros and plasma specimens on the Abaxis Piccolo for all analytes in the comprehensive metabolic panel.

**Fig. 3:**
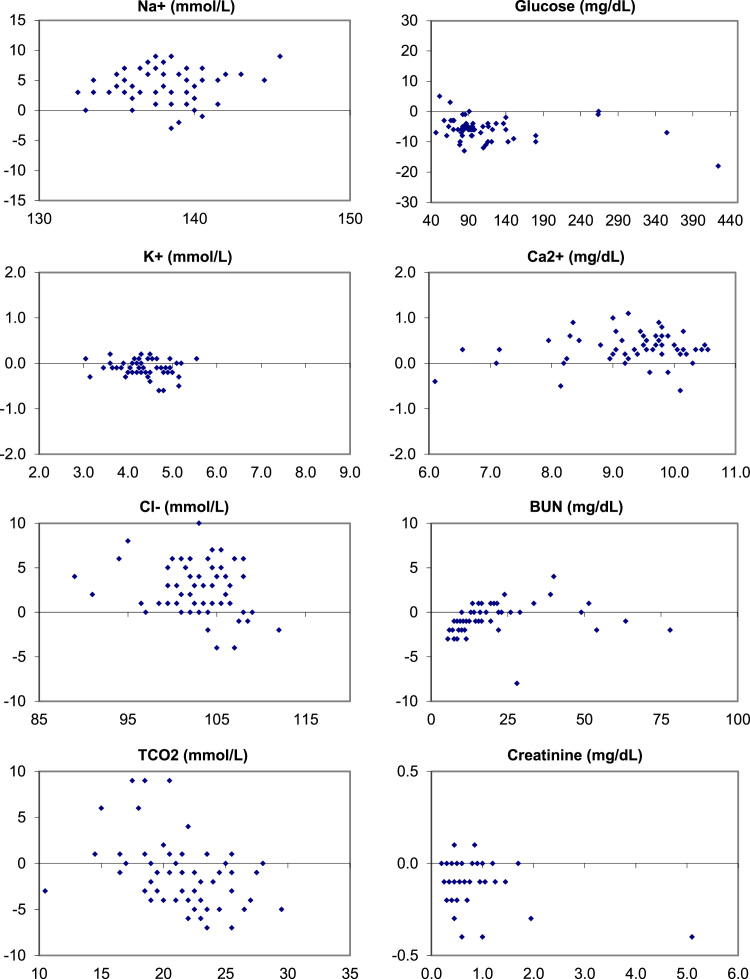

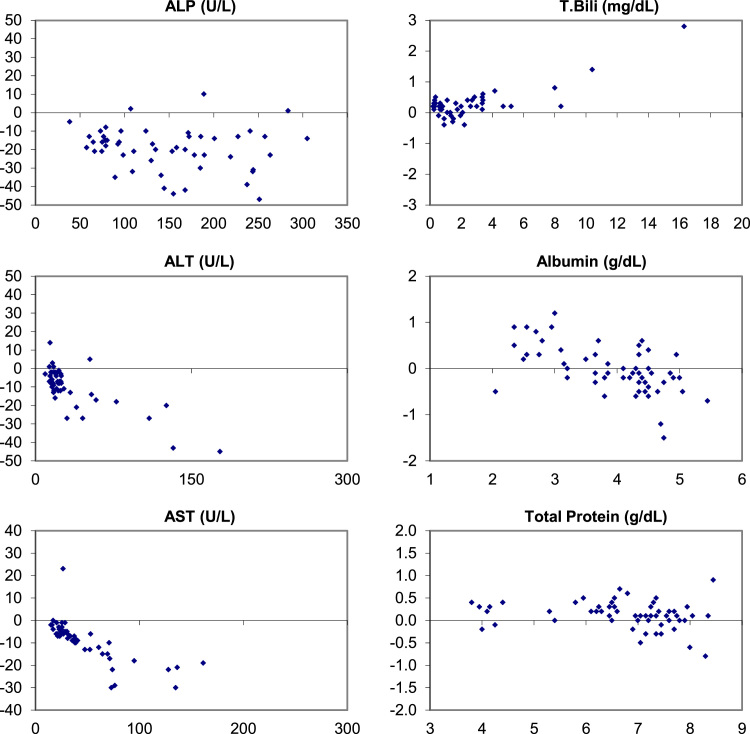
Bias (Bland-Altman) Plots of analytical bias between serum specimens on the Ortho Vitros and serum specimens on the Abaxis Piccolo for all analytes in the comprehensive metabolic panel.

The following data (Fig. 1, Fig. 2, Fig. 3) visually demonstrates the bias between the Ortho Vitros and the Abaxis Piccolo point of care device for each of the 14 comprehensive metabolic panel analytes for all three matrices tested: whole blood (Fig. 1), plasma (Fig. 2), and serum (Fig. 3).

## 2 Experimental design, materials and methods

Serum, plasma and whole blood samples for comparison testing between the Piccolo Xpress® (Abaxis, Union City, CA) and the Vitros® 5600 bio-analyzer (Ortho Clinical Diagnostics, Rochester NY) were obtained from waste specimens from the central laboratory at our institution. Sixty-five previously frozen serum samples drawn during routine clinical practice were thawed and immediately tested on both instruments. Sixty whole blood specimens were generated from residual samples from blood gas analysis drawn in lithium heparin syringes during the previous shift and tested immediately on both instruments. After whole blood testing the remaining sample was centrifuged and the resulting plasma was run on the Piccolo Xpress® and the Ortho Vitros®. All specimens were analyzed according to the manufacturer's instructions.

For this study, the Comprehensive Metabolic Reagent Disc containing a 14-chemistry panel consisting of sodium, potassium, chloride, calcium, carbon dioxide, BUN, creatinine, glucose, ALT, AST, alkaline phosphatase, total bilirubin, albumin and total protein, was used. Internal quality control wells on each disc address reagent quality, sample adequacy, optics, electronics and individual reaction parameters as well as interferences from lipemic, hemolytic or icteric specimens. In addition daily external quality control was run each day the instrument was in use.
